# Effects of Classical Music on Behavioral and Physiological Stress Markers in Socially Isolated Prairie Voles

**DOI:** 10.1111/nyas.70394

**Published:** 2026-09-03

**Authors:** Yessenia Chavez, Nathan Campbell, Yujun Liu, Hongdao Meng, Jamie Mayer, Christopher Wright, Alex Amidei, Insha Butail, Chloe Steffel, Chirantana Dayanandaswamy, Angela J. Grippo

**Affiliations:** ^1^ Department of Psychology Northern Illinois University DeKalb Illinois USA; ^2^ Gerontology/Leadership and Aging Services, School of Family and Consumer Sciences Northern Illinois University DeKalb Illinois USA; ^3^ School of Aging Studies University of South Florida Tampa Florida USA; ^4^ Speech‐Language Pathology, Division of Allied Health and Communicative Disorders Northern Illinois University DeKalb Illinois USA

**Keywords:** behavior, cardiovascular, classical music, corticosterone, prairie vole, social isolation

## Abstract

Chronic social stressors negatively affect behavioral, physiological, and neuroendocrine functioning. Interventions such as listening to music may alter behavioral and physiological responses to social stressors. This study investigated the effects of music exposure (vs. ambient noise) during chronic social isolation on physiological and behavioral outcomes in socially monogamous prairie voles. Potential sex differences in responses to chronic social isolation with and without music exposure were also examined. Music exposure significantly reduced validated anxiety‐ and depression‐related behavioral responses (increased exploration and grooming, and reduced immobility) and improved physiological measures (heart rate and heart rate variability) in chronically socially isolated prairie voles. Music exposure attenuated an endocrine indicator of stress relative to ambient noise conditions. Minor sex differences were observed; however, music protected against behavioral and physiological indicators of social isolation in both female and male prairie voles. This study demonstrates protective behavioral and physiological effects of music in a social context, and has translational potential for humans who experience chronic social isolation.

## Graphical abstract

Social isolation contributes to behavioral, physiological, and neuroendocrine dysfunction. In prairie voles, classical music exposure significantly reduced validated anxiety‐ and depression‐related behavioral responses and improved cardiac and endocrine measures as a function of chronic social isolation, with minor sex differences. This study demonstrates improved behavioral and physiological effects of music following chronic social isolation, with translational potential for socially isolated or lonely humans.

## 1 Introduction

Social stressors are highly complex adversities and include social isolation, feelings of loneliness, and negative or toxic social interactions. Social stressors are very prevalent, and loneliness and social isolation are experienced regardless of age, sex, socioeconomic status, and demographic variables [1, 2, 3]. As a recent example, it is estimated that over 50% of American adults reported feelings of loneliness during the COVID‐19 pandemic [4]. Although not equivalent to mood disorders, social isolation and loneliness contribute to the development of depression, anxiety, and additional emotion‐related disorders [5, 6, 7], thereby exhibiting significant negative effects on psychological and physiological health [8, 9]. The interactions of social stress with emotion and physiological consequences, such as elevated heart rate (HR) and blood pressure, can further exacerbate the emotional and physical toll associated with stress and contribute to the development of cardiovascular diseases [10, 11, 12, 13, 14, 15, 16]. Social stressors are also associated with altered endocrine processes, such as disrupted hypothalamic−pituitary−adrenal (HPA) axis function [17, 18]. Importantly, widespread social isolation and loneliness, coupled with significant negative consequences on psychological and physical health, prompted a 2023 US Surgeon General's Report, which characterized the lack of social connections as an epidemic [19].

With relevance to human health, animal models allow for the study of behavioral and physiological consequences of social stress and the specific influence of social and environmental stressors on neurobiological systems. For example, studies with nonhuman primates and rodents indicate that social isolation and social instability are associated with several behavioral changes relevant to stress, depression, and anxiety, as well as immune, endocrine, autonomic, and cardiovascular disturbances [1, 20, 21, 22, 23, 24, 25, 26]. Sex differences in response to social stressors have also been observed, for instance, in the cardiovascular profile of responses to witnessing social defeat in rats and in phenotypic behaviors relevant to depression in mice as a function of early life stress [24, 27].

In addition to studies with nonhuman primates and traditional laboratory rodent species, the prairie vole (*Microtus ochrogaster*) is a valuable rodent species for studying behavioral and physiological responses to social stressors. Both humans and prairie voles are socially monogamous mammals, exhibiting characteristic behaviors such as displaying an enduring pair bond with one partner, engaging in biparental and alloparental care of offspring, and helping conspecifics who are in distress [28, 29, 30]. In prairie voles, social bonds are critical for maintaining optimal health [28, 31, 32, 33]. Several forms of social stress in prairie voles significantly increase phenotypes indicative of depression and anxiety, while also decreasing parasympathetic input to the cardiovascular system, elevating stress hormone levels, and disrupting oxytocin and serotonin communication in stress‐relevant neural circuitry [34, 35, 36, 37, 38, 39]. These similarities to humans make prairie voles a valuable translational model to study social stress‐related behaviors and neurobiological processes. Some studies have also reported sex differences in prairie voles as a result of social stressors, including sex‐specific inflammatory, neuroendocrine, cardiovascular, and behavioral responses [40, 41, 42, 43]. These previous results suggest that evaluating sex differences in response to stress and social experiences in prairie voles can increase our understanding of these constructs in humans.

It is critical that we evaluate and understand feasible strategies to protect against negative consequences of social stress. Noninvasive, nonpharmacological treatments such as music listening have gained increasing attention as an approach for helping individuals cope with stress in several contexts. Listening to music shows considerable efficacy across various clinical contexts in humans. For example, music significantly reduces anxiety, accelerates stress recovery, and improves physiological markers of stress, including attenuating HR, HR variability, blood pressure, and cortisol levels [8, 9, 13, 44, 45]. The therapeutic applications of music extend beyond general stress reduction to specific populations with heightened stress vulnerability, such as in older individuals with Alzheimer's disease and in neonatal medical procedures, demonstrating music's capacity to improve psychological or physiological outcomes across diverse age groups [13, 46].

The neurobiological features that underlie the benefits of music may be homologous in humans and animals [47]. Animal models have thus offered valuable insight into music's potential benefits. For instance, animal studies involving both dogs and rodents have demonstrated that music can induce positive emotions and improve a breadth of otherwise adverse outcomes, such as attenuating depression‐ and anxiety‐related behaviors, decreasing stress hormones, improving HR variability over time, reducing hypertension, and improving outcomes associated with seizures and traumatic brain injury [48, 49, 50, 51, 52, 53, 54, 55, 56, 57]. It is possible that some of the benefits of music involve dopamine, gamma‐aminobutyric acid, glutamate, and/or brain‐derived neurotrophic factor mechanisms in the brain [52, 55, 58, 59, 60, 61, 62].

Whether music is beneficial in social contexts, and whether the benefits vary as a function of sex, requires further investigation. Although less understood than emotional and stress responses, it is possible that music improves social deficits and cognitive function in animal models, with some sex differences. Sex‐dependent effects of music on social behavior have been observed in nonhuman primates, where although music was associated with increased affiliative behavior in both sexes of captive chimpanzees, the effect was significantly more pronounced in females [63]. Research in a rat model of autism demonstrated some sex‐dependent changes in learning, memory, and sociability outcome measures following music exposure, with sex‐specific improvements interacting with developmental phases [60, 61]. Finally, in prairie voles exposed to a short‐term stress task, exposure to classical music reduced anxiety‐relevant behaviors and behavioral stress reactivity in socially isolated (but not in paired control) prairie voles, with minor sex differences [64]. Collectively, this previous research suggests that the evaluation of sex‐specific responses to social stress and music exposure will further inform our understanding of the translational benefits of music on behavioral and physiological health outcomes.

The current study was designed to investigate whether classical music would improve behavioral, cardiac, and endocrine responses to continuous, chronic social isolation in male and female prairie voles. Given that lyrics would not influence rodent behaviors and physiology in an analogous manner as they would in humans, classical music representing multiple tempos, keys, and rhythms was used in the present study. We hypothesized that intermittent exposure to classical music during a period of chronic social isolation would significantly improve specific behavioral and physiological outcome measures after the period of social isolation in both male and female prairie voles, including (1) behavioral responses related to depression and anxiety; (2) resting HR and HR variability responses; and (3) stress hormone metabolite levels. We further hypothesized that music exposure would have benefits for both sexes of prairie voles; and based on inconsistent responses to stress as well as environmental treatments across sexes noted above, we predicted that any sex differences observed in the outcome measures studied here would be minor.

## 2 Methods

### 2.1 Animals

Adult female and male prairie voles were studied across three experiments (*n* = 80; 60–120 days of age at the beginning of the experiment; 23–62 g body weight range), and all animals were naïve to any music prior to the experiment. Prairie voles were descendants of a stock originally captured near Champaign, IL. The prairie voles used in the present study were bred and housed in a research facility at Northern Illinois University (NIU). All animals were housed in family groups after birth. Weaning occurred on postnatal day 21, after which animals were housed in same‐sex sibling pairs. Standard housing conditions included housing in polycarbonate cages (12 × 18 × 28 cm) with ad libitum access to food (Purina rabbit chow), water, and bedding (Aspen Chip). Cotton nesting material was provided to all cages for enrichment (Ancare, Bellmore, NY). Animals were maintained on a 14/10 light/dark cycle (lights on at 6:30 am). The temperatures of the housing rooms were maintained between 24°C and 26°C, and relative humidity levels were maintained between 40% and 60%. All handling for cage changes and the measurement of body weight was standardized across groups and sexes throughout the experimental timeline. All procedures described here were approved by the NIU Institutional Animal Care and Use Committee; conducted according to the National Institutes of Health's (NIH) *Guide for the Care and Use of Laboratory Animals;* and followed all National Institutes of Health's 3Rs recommendations for reduction, refinement, and replacement. To ensure that the current study followed the 3Rs mandate, all prairie voles in the current study were socially isolated given previous evidence that additional environmental treatments (including music) do not alter behavior or physiology in prairie voles with appropriate social contact [64, 65, 66].

Sample sizes for each experiment described here were determined using power analyses based on preliminary data and previously published results [41, 43, 67, 68]. Effect sizes were calculated using Cohen's *d* values [69]. A probability value was set to *p* < 0.05 for the purpose of these analyses, with a desired power level of 0.80. These power analyses indicated that 6–8 animals per sex, per condition, were reasonable and justified for the procedures and analyses planned here. These results informed the sample sizes for experiments 1 and 2, which include 16 animals per condition (eight animals per sex, per condition). Group and sex comparisons are presented for all outcome measures in these first two experiments. Experiment 3 included eight animals per condition (four animals per sex, per condition); therefore, sex comparisons in this experiment are presented for exploratory purposes only.

### 2.2 General Study Design

The general design of social isolation and music versus ambient noise exposure across all experiments is described here. Specific procedures for each experiment are described in the following sections. A total of 80 prairie voles were used here (40 females and 40 males), with 32 prairie voles in experiment 1, 32 prairie voles in experiment 2, and 16 prairie voles in experiment 3.

#### 2.2.1 Social Isolation

All prairie voles (*n* = 80) were exposed to 8 weeks of continuous chronic social isolation during adulthood (age range 60–120 days at the beginning of the 8‐week paradigm). During this period, animals were continually housed in individual cages, separated from their previous same‐sex sibling, housed in a separate room without access to visual, olfactory, or auditory cues from the previous sibling. The housing room included standard ambient noise sounds of approximately 40 dB (range of 35–52 dB). The ambient noise in the housing room was produced from uncontrolled, variable environmental sounds such as fans cycling on or off as part of the heating, ventilation, and air conditioning system, personnel entering or leaving the room or conducting standard animal care duties in the room, or the muffled sounds of carts being moved in the hallway or doors opening or closing outside of the room. All animals were placed into clean cages once per week, with clean bedding, food, water, and cotton nesting material.

#### 2.2.2 Music Versus Ambient Noise Presentation

During the final 4 weeks of the chronic social isolation paradigm, prairie voles (*n* = 80 total) were divided into two experimental conditions that were determined a priori: exposure to either music (experimental; *n* = 20 males and 20 females) or ambient noise (control; *n* = 20 males and 20 females). All exposures to music or ambient noise occurred at the same time for each condition (approximately 4–6 h following light onset), in separate rooms, with standardized environmental conditions, and all animals remained in their isolated conditions during the exposures to music or ambient noise. Animals in the music condition were removed from the housing room and transported in the home cages into a separate room with continued access to food and water. A playlist of classical music clips was presented to the animals for approximately 30 min per session, twice per week, for a total of eight music exposure sessions (see Table 1 for list of song clips). Each playlist was unique, such that the animals did not hear the same playlist more than once. Music was played using an iPad set to a standardized mean volume of 60 dB (50–70 dB range based on the song), which was equidistant to all cages in the room. The music clips used here were chosen for the following characteristics: (1) the song clips did not contain any lyrics; (2) the song clips represented a variety of instruments, composers, tempos, keys, lengths, and volume shifts; and/or (3) the song clips have been shown to be associated with altered behavioral or physiological outcome measures in previous animal research experiments [52, 56, 64, 70].

**TABLE 1:** Song clips presented to the music exposure condition.

Song title and composer(s)	Performer(s), producer(s), and release information	Song clip length	Direct links to song clip or album
The Four Seasons Violin Concertos, No. 1 in E Major, Op. 8, RV 269 “Spring” (1718−1720), Antonio Vivaldi	Violin: Anne‐Sophie Mutter; Conductor: Herbert von Karajan; Producer: Peter Alward; Orchestra: Wiener Philharmoniker, 1999	11:11	https://www.discogs.com/master/420087‐Vivaldi‐Tartini‐Anne‐Sophie‐Mutter‐Trondheim‐Soloists‐The‐Four‐Seasons‐Devils‐Trill https://www.youtube.com/watch?v = nWmyIWwRI8g https://www.youtube.com/watch?v = 61Bj0lr4bPA https://www.youtube.com/watch?v = DCu0x536pOs
The Four Seasons Violin Concertos, No. 4 in F Minor, Op. 8, RV 297 “Winter” (1718−1720), Antonio Vivaldi	Violin: Anne‐Sophie Mutter; Conductor: Herbert von Karajan; Producer: Peter Alward; Orchestra: Wiener Philharmoniker, 1999	10:10	https://www.discogs.com/master/420087‐Vivaldi‐Tartini‐Anne‐Sophie‐Mutter‐Trondheim‐Soloists‐The‐Four‐Seasons‐Devils‐Trill https://www.youtube.com/watch?v = 5f_Y9wr46ig https://www.youtube.com/watch?v = XVGBaZkLEfE https://www.youtube.com/watch?v = _lsy5dpLfes
Meditation from Thaïs (1890), Jules Massenet	Violin: Anne‐Sophie Mutter; Producers: Roger Wright, Werner Mayer, Co‐producer: Ewald Markl, 1993	6:42	https://www.discogs.com/master/416395‐Anne‐Sophie‐Mutter‐Wiener‐Philharmoniker‐James‐Levine‐Carmen‐Fantasie https://www.youtube.com/watch?v = Aak_xByZntQ
Piano Sonata No. 2 in B‐flat Minor, Op. 35 I. Grave—Doppio Movimento (1838), Frédéric Chopin	Piano: Maurizio Pollini; Producer: Rainer Brock, 1985	7:17	https://www.youtube.com/watch?v=KAgHumUlYv0
Concerto for Piano and Orchestra No. 1 in E Minor, Op. 11 – II. Romance Larghetto (1830), Frédéric Chopin	Piano: Zubin Mehta, Lang Lang; Producers: Christian Leins, Christopher Alder, 2008	10:00	https://www.youtube.com/watch?v=7xcjgSGywpo
Sonata for Two Pianos in D Major, K.448 375a I. Allegro con Spirito (1781), Wolfgang Amadeus Mozart	Piano: Zoltán Kocsis, Dezső Ránki, 1987	7:45	https://www.youtube.com/watch?v = E7SS2BXSPj8
Barcarolle in F‐Sharp Major, Op.60, Frédéric Chopin	Piano: Maurizio Pollini; Producers: Christopher Adler, Werner Mayer, 1991	8:33	https://www.youtube.com/watch?v=CztOXaox2nA
Piano Concerto No. 6 in B Flat, K.238: iii. Rondo Allegro, Wolfgang Amadeus Mozart	Piano: Jeno Jando; Label: Children's Group, 2000	6:45	https://www.amazon.com/Music‐Children‐4‐Mozart‐Go/dp/B00004X0Q8 https://www.youtube.com/watch?v = CutRL28‐9x8
Polonaise‐Fantasie in A‐Flat Major, Op. 61, Frédéric Chopin	Piano: Maurizio Pollini; Producer: Rainer Brock, 2010	13:08	https://www.youtube.com/watch?v=uZp983JSOFo
Piano Sonata No. 11 in A Major, K.331: iii. Rondo Alla Turca, Wolfgang Amadeus Mozart	Piano: Yondo; Label: Warner Classics, 2024	3:23	https://www.youtube.com/watch?v=n2Tru4Qa3pc

*Note*: Song clips were arranged in eight unique playlists, with each playlist containing approximately 30 min of music. Song clips were acquired from YouTube or from prepurchased CDs. A link to each clip is provided in this table; for a song acquired from a CD, the link to the album is provided along with a publicly available sample posted on YouTube.

Animals in the ambient noise condition were removed from the housing room and transported in the home cages into a separate room at the same time as the music‐exposed condition (approximately 4–6 h following light onset) for the same duration as the music exposure, with continued access to food and water. The environmental conditions in the ambient noise condition were uncontrolled and variable, including environmental sounds from the heating/cooling fan or return air vent in the room, the muffled sound of a cart being moved in the hallway outside the room, or the muffled sound of a door opening or closing outside of the room. The mean volume level of the ambient noise was 40 dB (35–47 dB range). Following the music exposure or ambient noise conditions, all cages were returned to the housing rooms at the same time.

#### 2.2.3 Body Weight Measurements

Body weight was measured in all animals prior to social isolation (baseline), following 4 weeks of chronic social isolation, and following 4 weeks of continued chronic isolation with either music or ambient noise exposure.

### 2.3 Specific Experimental Procedures

#### 2.3.1 Experiment 1: Behavioral Measures

In experiment 1, behaviors were evaluated across two operational tasks in a subset of male and female prairie voles in each condition (*n* = 32 prairie voles total; *n* = 16 animals per condition; *n* = 8/sex/condition). Twenty‐four hours following the 8‐week period of chronic social isolation and music/ambient noise exposure, and approximately 4–6 h following light onset, each animal was exposed to a 5‐min swim task for the evaluation of passive versus active behaviors during a short‐term stressor [38, 43, 71, 72, 73]. The apparatus consisted of a cylindrical Plexiglas tank (46 cm height; 20 cm diameter), filled to a depth of 18 cm with tap water (room temperature, approximately 24°C–26°C). Each animal was placed gently into the tank and allowed to move freely for a total of 5 min, during which time behaviors were recorded from the side using a digital video camera (Canon USA, Inc., Melville, NY). The animal was gently removed from the tank immediately following the 5‐min trial and returned to the home cage. A heat lamp was provided for 15 min to ensure the recovery of body temperature. The tank was cleaned with water and diluted bleach, then filled with clean water, prior to testing each animal.

Behaviors during the swim task were coded off‐line by three experimentally blind observers who were trained to a level of at least 90% inter‐rater reliability. Each experimenter coded the following behaviors using standardized operational definitions: (1) *swimming*, including coordinate movements of the forelimbs and hindlimbs as the animal moved around in the water; (2) *struggling*, including uncoordinated movements of the forelimbs, which break the surface of the water in the middle of the tank; (3) *climbing*, including uncoordinated movement of the forelimbs, which break the surface of the water at the wall of the apparatus; and (4) *immobility*, including complete lack of movement or minor movements of the forelimbs and/or hindlimbs to remain afloat, without breaking the surface of the water and without trunk movements or a physical change in location in the water. The durations of swimming, struggling, and climbing were summed to provide an index of active coping behaviors. The duration of immobility was categorized as an index of a passive, maladaptive response to the stressor, validated in previous research in the prairie vole model to represent a state of stress [34, 43, 73]. The categories of adaptive plus maladaptive behaviors are exhaustive, mutually exclusive, and comprise the entire 5 min swim trial.

Forty‐eight hours following the swim task, each animal was exposed to a 5‐min open field test (OFT), approximately 4–6 h following light onset, conducted in a brightly lit room, for the evaluation of behavioral stress responses and exploration [74, 75, 76, 77, 78]. The OFT apparatus consisted of a square chamber (40 cm width; 40 cm length; 40 cm height) with an inner square marked with red tape (10 × 10 cm) noting a separation of the center versus the surround of the apparatus. Each animal was placed gently into the apparatus and allowed to explore freely for a total of 5 min, during which time behaviors were recorded from the top using a digital video camera (Canon USA, Inc.). The animal was immediately removed gently at the end of the 5‐min trial and replaced in the home cage. The chamber was cleaned with water and diluted bleach solution, then dried thoroughly, prior to testing each animal.

Behaviors during the OFT were coded off‐line by three experimentally blind observers who were trained to a level of at least 90% inter‐rater reliability. Each experimenter coded the following behaviors using standardized operational definitions: (1) *duration of exploration of the center section*, requiring all four paws to be physically inside the center section; (2) *duration of grooming*, including cleaning or scratching of one's fur with the mouth or paws; (3) *duration of exploratory motion*, requiring horizontal movement involving a physical change in location; vertical motion and circular motion without a physical change in location were excluded from this category); (4) *frequency of rearing*, including lifting of the front paws off the ground followed by replacing the paws on the ground; this behavior may occur in the center or the surround section of the apparatus or at the wall or corner; and (5) *frequency of escape attempts*, including jumping at the wall or corner of the apparatus, scratching or chewing in areas where the walls are connected or where the walls meet the floor, or chewing, scratching, or pulling on the metal bars that hold the walls in place [74, 75, 76, 77, 78, 79, 80, 81, 82].

#### 2.3.2 Experiment 2: Cardiac Measures

In experiment 2, cardiac indices were evaluated across three measures in a subset of male and female prairie voles in each condition (*n* = 32 animals total; *n* = 16 animals per condition; *n* = 8/sex/condition). Approximately 72 h following the 8‐week period of chronic social isolation and music/ambient noise exposure, and approximately 4–6 h following light onset, each animal was anesthetized with isoflurane (3%–4%; Henry Schein, Dublin, OH) mixed with oxygen. A wireless radiofrequency transmitter (TA10ETA‐F10; Data Sciences International, St. Paul, MN) was surgically implanted for continuous recording of the electrocardiogram (ECG) with DII electrode placement on either side of the heart, using procedures described previously [25, 43, 83, 84].

A 10‐min recording of ECG was conducted using a radiotelemetry receiver and associated software to obtain a sample of resting ECG without movement confounds (Dataquest ART Acquisition, Data Sciences International; sampling rate 5 kHz, 12‐bit precision digitizing). The amount, delivery rate, and specific time course of isoflurane and oxygen were controlled across all animals to ensure standardized recording conditions, with preliminary validation experiments and previously published procedures indicating no statistical difference between an anesthetized ECG recording and a recording under resting basal conscious conditions (with movement artifact excluded) in the prairie vole [43].

All ECG data were manually inspected off‐line for appropriate signal‐to‐noise ratio by trained, experimentally blind experimenters. HR was quantified as the number of beats per minute (bpm) using the vendor software. HR variability was quantified from the R‐R intervals using procedures described previously [43, 85, 86, 87], including standard deviation of all normal‐to‐normal intervals (SDNN index), which is hypothesized to reflect sympathetic and parasympathetic convergence on the cardiac pacemaker; and root mean square of successive differences between normal heartbeats (RMSSD), which is hypothesized to represent HR alterations as a function of parasympathetic communication with the heart.

#### 2.3.3 Experiment 3: Endocrine Measure

In experiment 3, endocrine indices were evaluated across time in a subset of male and female prairie voles in each condition (*n* = 16 animals total; *n* = 8 animals per condition; *n* = 4/sex/condition). Fecal samples were collected at three time points throughout the experiment, approximately 4–6 h following light onset, using procedures described previously: prior to social isolation (baseline); following 4 weeks of chronic social isolation; and following 4 weeks of continued chronic isolation with either music or ambient noise exposure (approximately 72 h after the end of the 4‐week period) [67, 88, 89]. For each collection date, each animal was placed into a new, clean cage, 24 h prior to the sample collection. Fecal pellets were collected from the cage, placed into a labeled collection vial, and stored at −20°C until assayed for corticosterone metabolites.

Fecal samples from each time point were prepared for the corticosterone metabolite assay using procedures described previously [67, 88, 89], as well as initial validation procedures, and conducted on ice to minimize hormone degradation. A standard mass of 0.05 g of fecal material was weighed and placed into a labeled collection tube, followed by homogenization. To extract corticosterone metabolites, 80% methanol was added to each sample, and the sample was agitated on a microplate shaker for 30 min. Each sample was subsequently centrifuged at 2500 rpm for 15 min. The supernatant was collected and placed into a new, labeled tube, and the sample was diluted at a ratio of 1:5 with assay buffer. Corticosterone metabolites were quantified in diluted samples with a commercially available corticosterone/metabolite ELISA kit according to the kit instructions (Enzo Life Sciences, Farmingdale, NY). Each sample was assayed in duplicate, and the intra‐ and inter‐assay coefficients of variance were each less than 5%.

### 2.4 Data Analyses

Data from each outcome measure were analyzed using independent groups or mixed‐design analyses of variance, followed by hypothesis‐driven and statistically justified Student's *t*‐tests, with a Bonferroni correction applied to all multiple comparisons (SPSS Statistics Version 29, IBM, Armonk, NY). A probability level of *p* < 0.05 was used to define statistically significant effects for initial hypothesis testing. For all pairwise comparisons involving a Bonferroni correction, the probability value was defined as statistically significant if it exceeded the adjusted probability value based on the number of pairwise comparisons conducted. Effect sizes are represented by Cohen's *d* values 69. The data did not violate assumptions of normality or unequal variances, with a few exceptions of the latter (noted throughout the Results section). When the variances were not equal, statistical tests assuming unequal variances are reported.

Because the groups for the experimental manipulation (music or ambient noise exposure) were determined a priori for each experiment, the following group names are used consistently throughout the presentation of results and figures: sex (male vs. female), group (music vs. ambient noise exposure), and time (experiment 3 outcome measures and measurement of body weight only; baseline vs. following 4 weeks of chronic social isolation vs. following 4 additional week of continued isolation with either music or ambient noise exposure).

## 3 Results

### 3.1 Experiment 1: Behavioral Results

**FIGURE 1:**
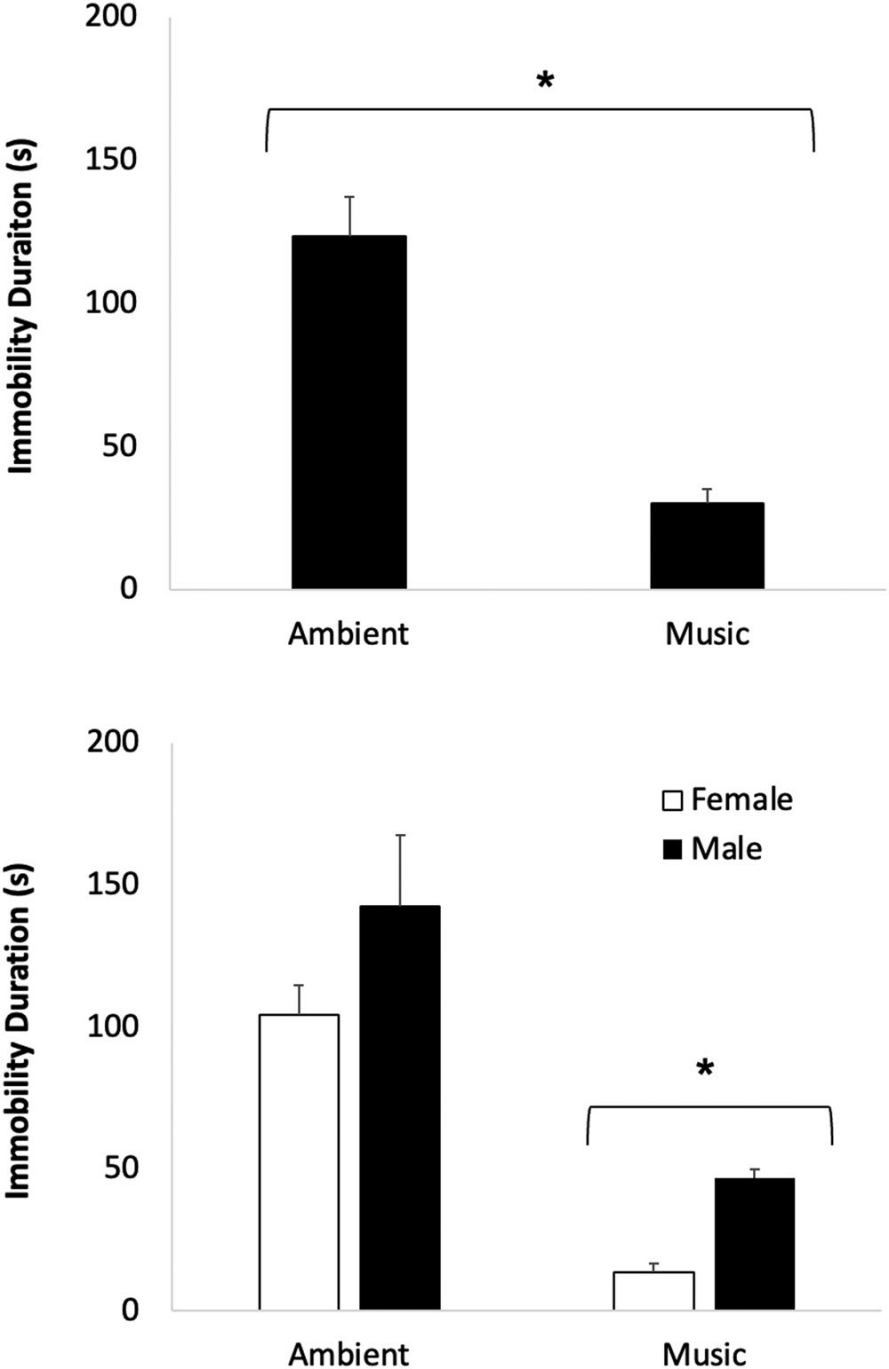
Immobility in the swim task in experiment 1. (A) Mean (+ SEM) duration of immobility in the swim task in prairie voles following 4 weeks of chronic social isolation and an additional 4 weeks of continued isolation with either music or ambient noise exposure (sexes collapsed). (B) Mean (+ SEM) duration of immobility in the conditions separated by sex. Females exposed to music displayed lower immobility values than males exposed to music. No sex difference was observed in the ambient noise condition. *p < 0.05 for the comparison indicated. *Note*: The remainder of the 5 min of the swim trial is comprised of summed active behavioral responses (swimming, struggling, climbing).

Immobility duration levels in the swim task are shown in Figure 1 (the remainder of the 5 min trial in this figure is represented by the summed durations of swimming, struggling, and climbing). Using a two‐way independent groups ANOVA, main effects of each sex (*F*(1,32) = 7.0, *p* < 0.01) and group (music or ambient noise exposure) (*F*(1,32) = 46.8, *p* < 0.001) were observed. No sex by group interaction was observed (*p* > 0.05). With sexes pooled, an independent groups Student's *t*‐test assuming unequal variances indicated that the music exposure condition displayed significantly lower immobility values than the ambient noise condition (*t*(30) = 6.3, *p* < 0.0001, *d* = 2.3). Additional *t*‐tests using a Bonferroni correction were conducted to evaluate possible sex differences. Immobility values did not differ by sex in the ambient noise condition (*p* > 0.05). Immobility values were significantly lower in the female music exposure condition relative to the male music exposure condition (*t*(14) = 7.3, *p* < 0.0001, *d* = 3.6).

**FIGURE 2:**
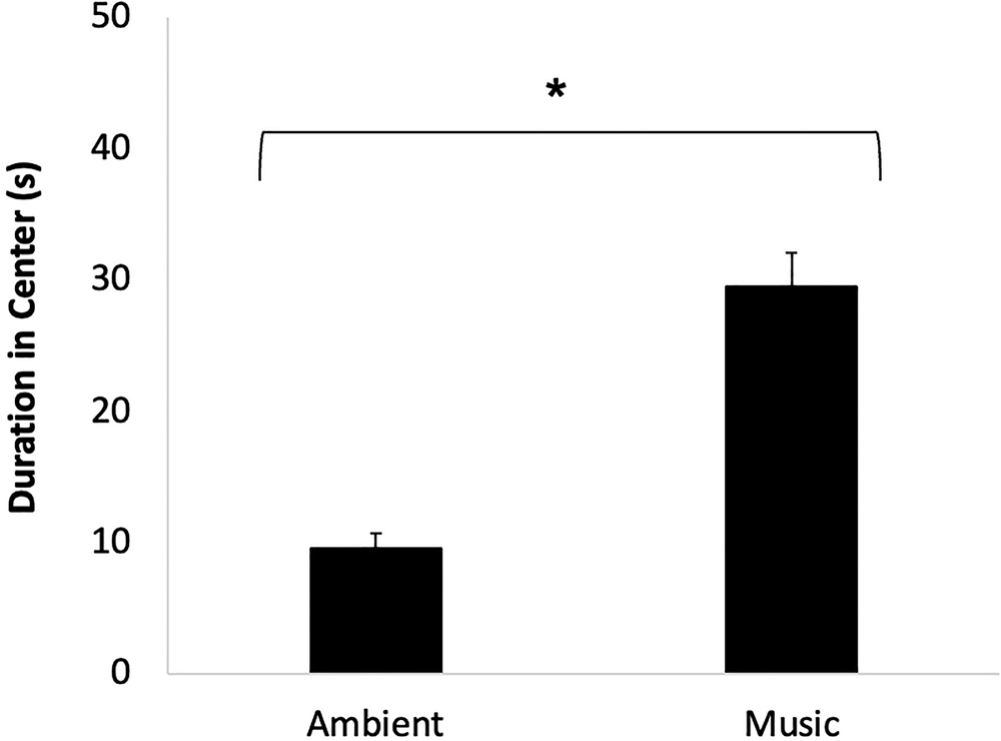
Center section exploration in the OFT in experiment 1. Mean (+ SEM) duration spent exploring the center of the OFT in prairie voles following 4 weeks of chronic social isolation and an additional 4 weeks of continued isolation with either music or ambient noise exposure (sexes collapsed). Prairie voles exposed to music displayed a greater duration of center section exploration than prairie voles exposed to ambient noise conditions. No sex differences were observed. *p < 0.05.

Duration of center section exploration in the OFT is shown in Figure 2. Using a two‐way independent groups ANOVA, a main effect of group (music or ambient noise exposure) was observed (*F*(1,32) = 29.4, *p* < 0.001). No main effect of sex or sex by group interaction was observed (*p* > 0.05 for both comparisons). With sexes pooled, an independent groups Student's *t*‐test assuming unequal variances indicated that the music exposure condition displayed significantly higher duration of exploration of the center section than the ambient noise condition (*t*(30) = 5.2, *p* < 0.0001, *d* = 1.9).

**FIGURE 3:**
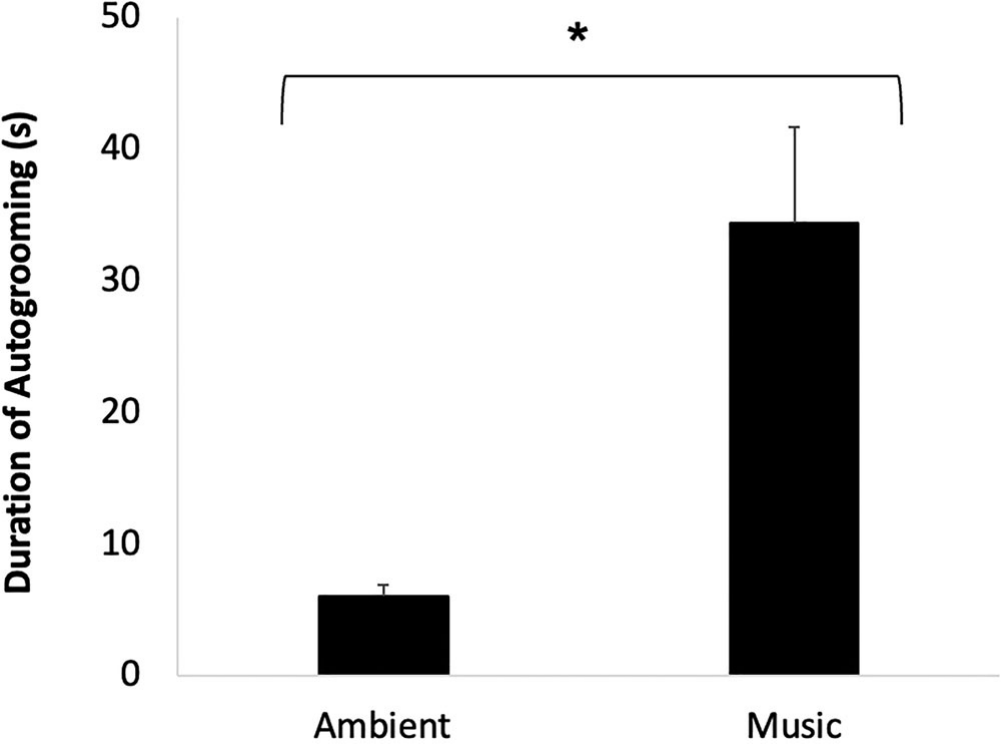
Autogrooming in the OFT in experiment 1. Mean (+ SEM) duration spent autogrooming in the OFT in prairie voles following 4 weeks of chronic social isolation and an additional 4 weeks of continued isolation with either music or ambient noise exposure (sexes collapsed). Prairie voles exposed to music displayed a greater duration of autogrooming than prairie voles exposed to ambient noise conditions. No sex differences were observed. *p < 0.05.

Duration of grooming in the OFT is shown in Figure 3. Using a two‐way independent groups ANOVA, a main effect of group (music or ambient noise exposure) was observed (*F*(1,32) = 15.4, *p* < 0.001). No main effect of sex or sex by group interaction was observed (*p* > 0.05 for both comparisons). With sexes pooled, an independent groups Student's *t*‐test assuming unequal variances indicated that the music exposure condition displayed significantly higher levels of grooming duration than the ambient noise condition (*t*(15) = 3.9, *p* < 0.002, *d* = 1.4).

No significant main effects of sex or group, or sex by group interactions, were observed for the following behaviors in the OFT: duration of motion, frequency of rearing, and frequency of escape attempts (*p* > 0.05 for all comparisons). No follow‐up tests were conducted (data not shown).

### 3.2 Experiment 2: Cardiac Results

**FIGURE 4:**
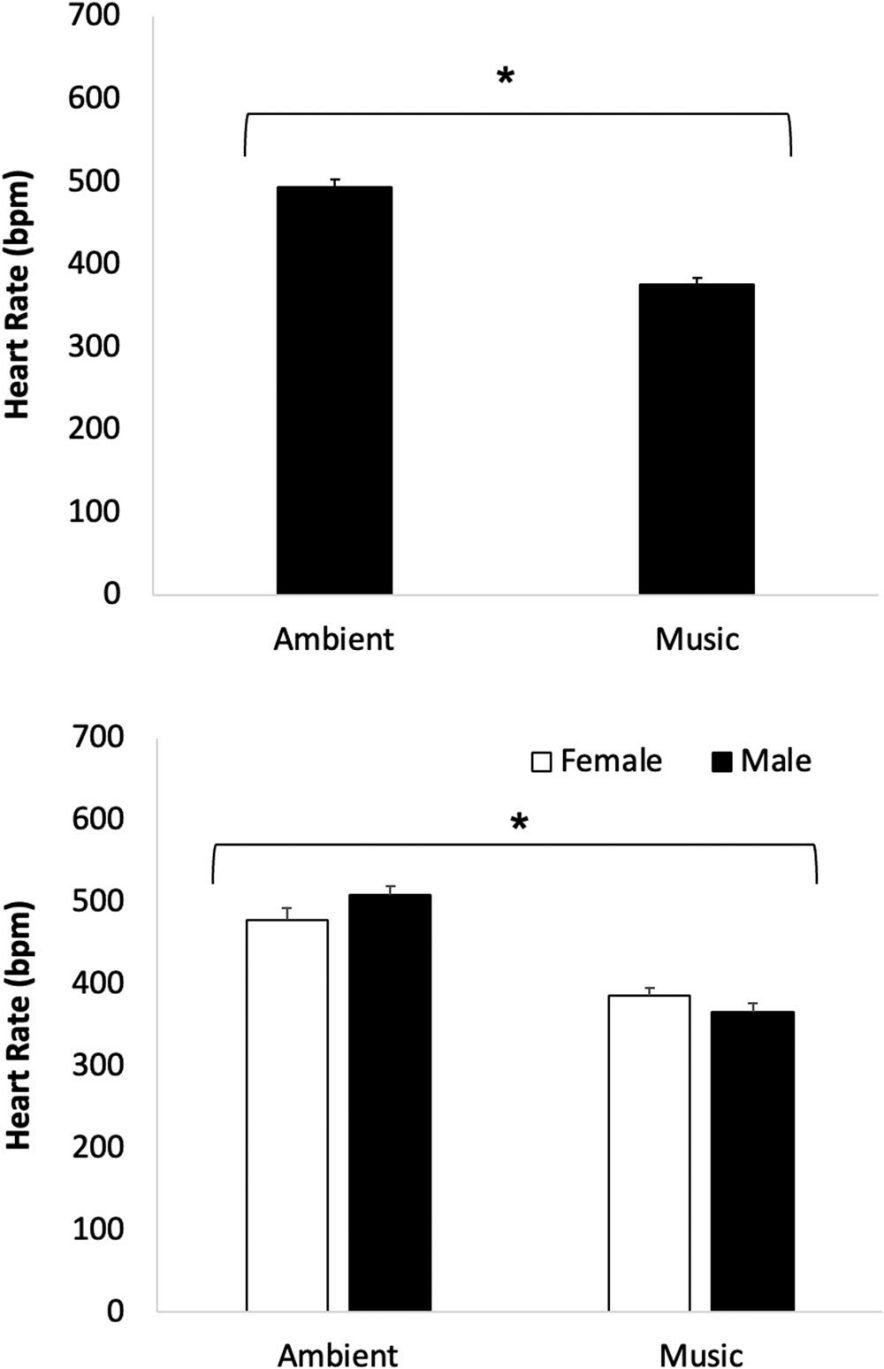
Resting HR in experiment 2. (A) Mean (+ SEM) resting HR in prairie voles following 4 weeks of chronic social isolation and an additional 4 weeks of continued isolation with either music or ambient noise exposure (sexes collapsed). (B) Mean (+ SEM) resting HR in each condition separated by sex. Both females and males in the music exposure conditions displayed lower resting HR values relative to the same sex in the ambient noise condition, with a stronger difference noted in males. *p < 0.05 for the comparison indicated.

Resting HR is shown in Figure 4. Using a two‐way independent groups ANOVA, a main effect of group (music or ambient noise exposure) was observed (*F*(1,32) = 99.6, *p* < 0.001), and a minor sex by group interaction (*F*(1,32) = 4.5, *p* < 0.04). No main effect of sex was observed (*p* > 0.05). With sexes pooled, an independent groups Student's *t*‐test assuming unequal variances indicated that the music exposure condition displayed significantly lower HR than the ambient noise condition (*t*(30) = 9.6, *p* < 0.0001, *d* = 3.4). Additional *t*‐tests using a Bonferroni correction were conducted to evaluate possible sex differences. HR was significantly lower in females in the music exposure condition relative to females in the ambient noise condition (*t*(14) = 5.3, *p* < 0.0001, *d* = 2.7); and HR was significantly lower in males in the music exposure condition versus males in the ambient noise condition (*t*(14) = 9.0, *p* < 0.0001, *d* = 4.5), with a stronger effect in males.

**FIGURE 5:**
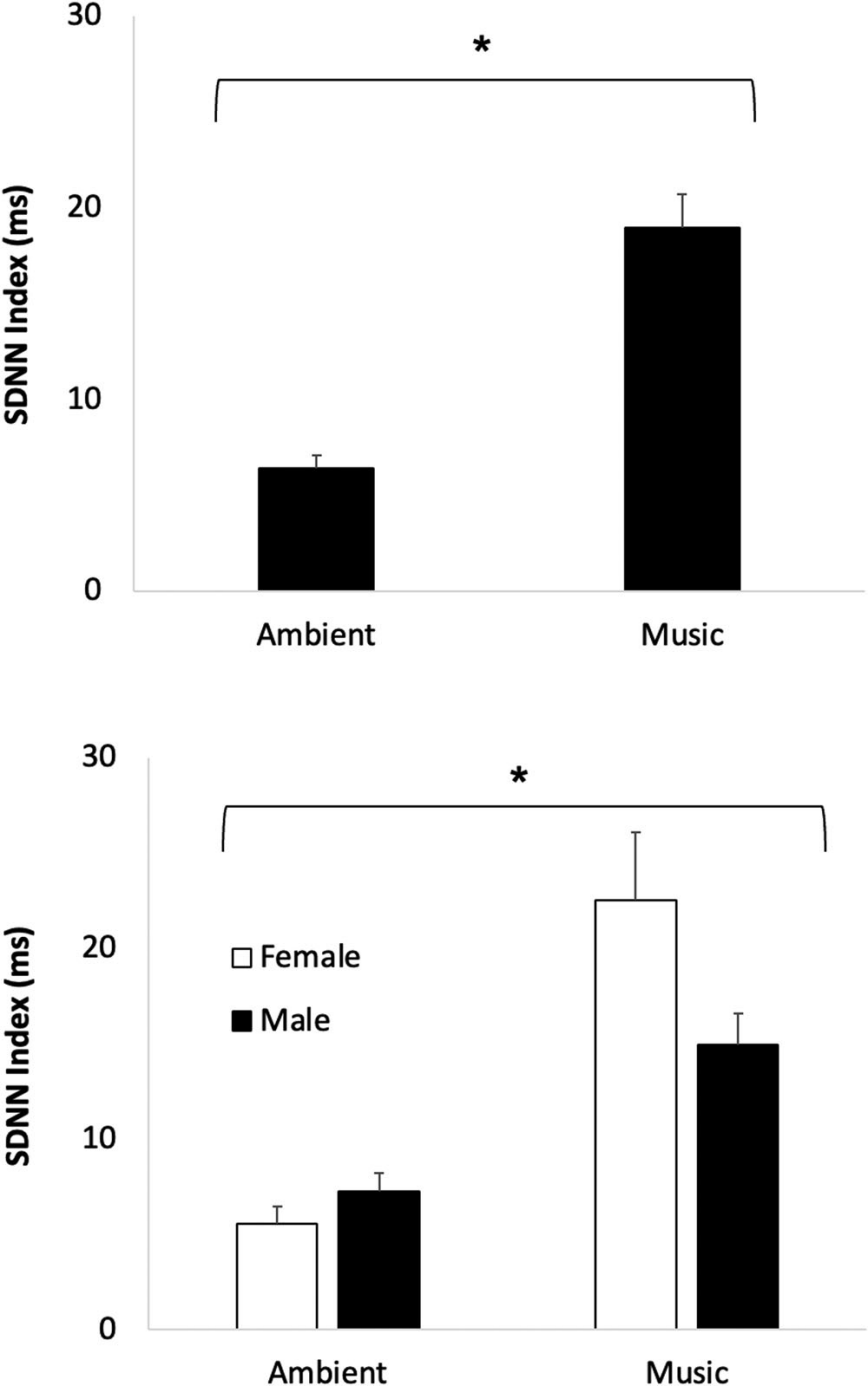
Resting SDNN index in experiment 2. (A) Mean (+ SEM) resting SDNN index in prairie voles following 4 weeks of chronic social isolation and an additional 4 weeks of continued isolation with either music or ambient noise exposure (sexes collapsed). (B) Mean (+ SEM) SDNN index in each condition separated by sex. Females in the music condition displayed a higher SDNN index versus females in the ambient noise condition; males in the music condition displayed a higher SDNN index versus males in the ambient noise condition, with similar effect sizes but a slightly stronger effect noted in females. *p < 0.05 for the comparison indicated.

Resting SDNN index is shown in Figure 5. Using a two‐way independent groups ANOVA, a main effect of group (music or ambient noise exposure) was observed (*F*(1,32) = 36.0.6, *p* < 0.001), and a minor sex by group interaction (*F*(1,32) = 5.1, *p* < 0.03). No main effect of sex was observed (*p* > 0.05). With sexes pooled, an independent groups Student's *t*‐test indicated that the music exposure condition displayed a significantly higher SDNN index than the ambient noise condition (*t*(30) = 5.5, *p* < 0.0001, *d* = 2.0). Additional comparisons, separated by sex with a Bonferroni correction applied, demonstrated that females in the music condition displayed a higher SDNN index versus females in the ambient noise condition (*t*(14) = 4.7, *p* < 0.0004, *d* = 2.4); and males in the music exposure condition displayed a higher SDNN index versus males in the ambient noise condition (*t*(14) = 4.0, *p* < 0.001, *d* = 1.9), with similar effect sizes (but a slightly stronger effect in females).

**FIGURE 6:**
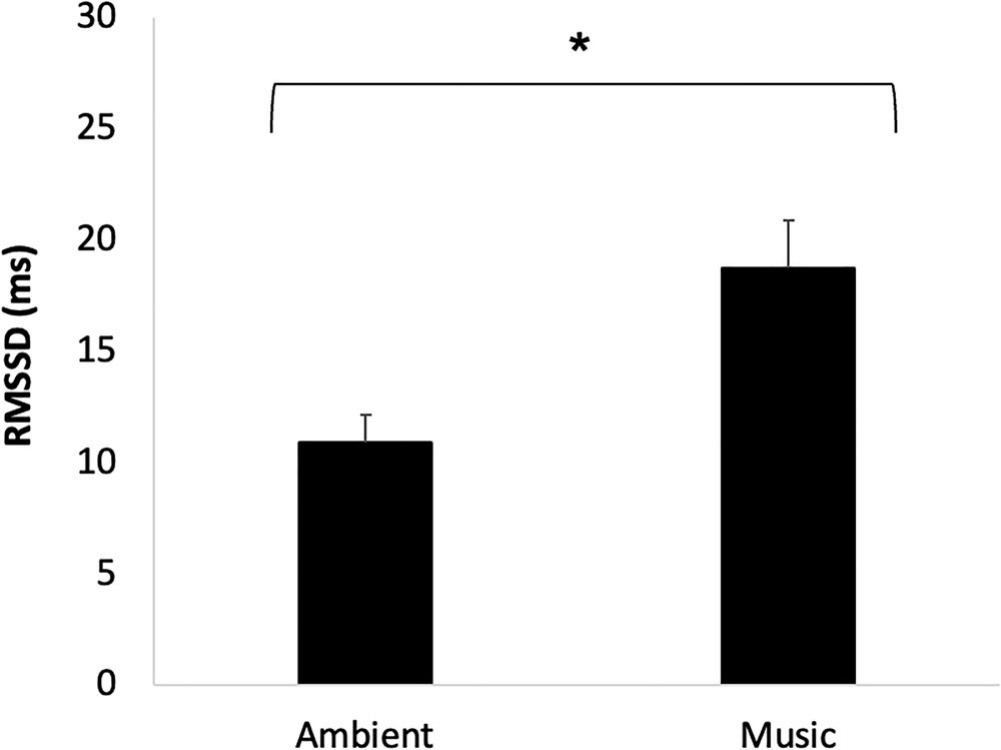
Resting RMSSD in experiment 2. Mean (+ SEM) resting RMSSD in prairie voles following 4 weeks of chronic social isolation and an additional 4 weeks of continued isolation with either music or ambient noise exposure (sexes collapsed). Prairie voles exposed to music displayed a greater RMSSD than prairie voles exposed to ambient noise conditions. No sex differences were observed. *p < 0.05.

Resting RMSSD is shown in Figure 6. Using a two‐way independent groups ANOVA, a main effect of group (music or ambient noise exposure) was observed (*F*(1,32) = 13.6, *p* < 0.001). No main effect of sex or sex by group interaction was observed (*p* > 0.05 for both comparisons). With sexes pooled, an independent groups Student's *t*‐test indicated that the music exposure condition displayed a significantly higher RMSSD than the ambient noise condition (*t*(30) = 3.7, *p* < 0.0008, *d* = 1.3).

### 3.3 Experiment 3: Endocrine Results

**FIGURE 7:**
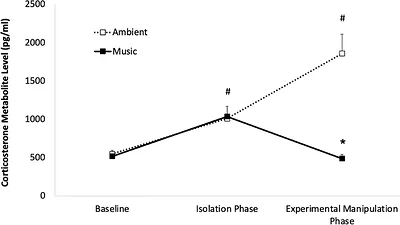
Corticosterone metabolite levels across time in experiment 3. Mean (+ SEM) corticosterone metabolite levels prior to social isolation (baseline), following 4 weeks of social isolation (isolation phase), and following 4 weeks of additional social isolation with either music or ambient noise exposure (experimental manipulation phase). Groups for the experimental manipulation phase were determined a priori; however, all animals received the same conditions at baseline and during the isolation phase of the design. No baseline differences were observed between ambient‐ and music‐exposed conditions (*p* > 0.05), as expected. Both conditions displayed a similar significant increase in corticosterone metabolite levels following 4 weeks of social isolation, relative to respective baseline levels, as expected. The ambient noise condition continued to display significantly higher corticosterone metabolite levels following the experimental manipulation phase, relative to this condition's respective baseline and social isolation levels. The music‐exposed condition displayed significantly lower corticosterone metabolite levels relative to the ambient noise condition following the experimental manipulation phase. Corticosterone metabolite levels in the music‐exposed group did not significantly differ from this condition's respective baseline value following the experimental manipulation phase, and levels were significantly lower than this condition's respective social isolation value. No sex comparisons were conducted. ^#^ *p* < 0.05 versus respective condition's baseline value; *p < 0.05 versus the ambient noise condition at the same time point.

Corticosterone metabolite levels are shown across time in Figure 7. The groups for the experimental manipulation (music vs. ambient noise exposure) were determined a priori; therefore, the group names are used consistently throughout the results and in Figure 7 despite the groups experiencing the same conditions at baseline and following 4 weeks of chronic social isolation, before the beginning of music or ambient noise exposure. A mixed‐design ANOVA, with sex (male vs. female) and group (music vs. ambient noise exposure) as independent factors and time (baseline in all animals, following 4 weeks of social isolation in all animals, following 4 additional weeks of continued chronic social isolation with either music or ambient noise exposure) as a repeated factor, yielded a main effect of group (music or ambient noise exposure) (*F*(1,12) = 21.0, *p* < 0.001) and a main effect of time (*F*(2,12) = 22.5, *p* < 0.001), with no main effect of sex (*p* > 0.05). A significant group by time interaction was observed (*F*(1,12) = 24.4, *p* < 0.001), with no significant sex by group interaction, sex by time interaction, or three‐way interaction (sex by group by time) (*p* > 0.05 for all comparisons).

Independent groups *t*‐tests with sexes collapsed, with a Bonferroni correction applied, indicated no significant group difference in corticosterone metabolite levels between the ambient noise and music exposure conditions at baseline or following social isolation (*p* > 0.05 for both comparisons). The music exposure condition displayed significantly lower corticosterone metabolite levels relative to the ambient noise condition following the experimental manipulation of the groups (music or ambient noise exposure) (*t*(14) = 5.3, *p* < 0.0001, *d* = 2.6).

Repeated measures *t*‐tests with sexes collapsed, with a Bonferroni correction applied, indicate the following. (1) Corticosterone metabolite levels following social isolation were significantly higher than baseline levels in both the ambient noise condition (*t*(7) = 6.0, *p* < 0.0005, *d* = 3.2) and the music exposure condition (*t*(7) = 4.4, *p* < 0.0003, *d* = 1.8), with a stronger effect in the ambient noise condition. (2) Corticosterone metabolite levels following the experimental manipulation of the groups (music or ambient noise exposure) were significantly higher than those following social isolation in the ambient noise condition (*t*(7) = 3.1, *p* < 0.01, *d* = 1.6), but were significantly lower than those following social isolation in the music exposure condition (*t*(7) = 3.7, *p* < 0.008, *d* = 1.9), with similar effect sizes. (3) Corticosterone metabolite levels following the experimental manipulation of the groups (music or ambient noise exposure) were significantly higher than baseline levels in the ambient noise condition only (*t*(7) = 5.2, *p* < 0.001, *d* = 2.5), but did not significantly differ from baseline levels in the music exposure condition (*p* > 0.05).

### 3.4 Body Weight

Across all experiments, male prairie voles were heavier than female prairie voles at baseline (male body weight = 43 ± 1 g, female body weight = 34 ± 1 g, *t*(78) = 6.4, *p* < 0.0001); following 4 weeks of chronic social isolation (male body weight = 44 ± 1 g, female body weight = 36 ± 1 g; *t*(78) = 5.3, *p* < 0.0001); and following 4 additional weeks of continued isolation with either music or ambient noise exposure (male body weight = 44 ± 1 g, female body weight = 36 ± 1 g; *t*(78) = 5.4, *p* < 0.0001). Body weight did not differ as a function of group (music or ambient noise condition) or time (baseline vs. following 4 weeks of chronic social isolation vs. following 4 additional weeks of continued isolation with either music or ambient noise exposure) (*p* > 0.05 for all comparisons; data not shown).

## 4 Discussion

Social isolation and loneliness are critical public health concerns [3, 19]. It is, therefore, essential that we understand behavioral and physiological processes related to specific prevention and treatment strategies for chronic social stressors. In particular, listening to music is a feasible and accessible strategy for improving stress‐relevant outcomes, but the sex‐specific benefits of music in a social context are less well understood. The current study demonstrated several positive protective effects of listening to music against consequences of chronic social isolation in both male and female prairie voles, including behavioral, cardiac, and endocrine outcomes. Across three experiments, this research provides evidence that listening to classical music during chronic social isolation significantly improves later behaviors related to depression and anxiety, improves indices of cardiac function including reduced HR and increased HR variability, and reduces corticosterone metabolite concentrations to a level similar to baseline levels. Although some minor sex differences were noted here, perhaps indicating that females (vs. males) show greater behavioral improvements relevant to depression in response to music, but that males (vs. females) show greater cardiac improvements in some measures, many of the outcomes suggest that listening to music benefits both sexes. This research has translational implications for understanding the benefits of music listening in humans who experience chronic loneliness and social isolation, and provides a foundation for evaluating sex‐specific neural mechanisms that underlie the protective influence of listening to music.

Extending previous research [52, 56, 90, 91] into a social context, the current study observed that listening to music during a period of chronic social isolation has beneficial effects on later behavioral outcome measures. In experiment 1, music exposure during chronic social isolation promoted adaptive emotion‐related behavioral responses in both males and females, whereas prairie voles exposed to control conditions (ambient noise) during chronic social isolation displayed maladaptive responses to the stress‐inducing environments. Specifically, during the swim task, previous music exposure reduced immobility levels in both sexes, relative to ambient noise. Music‐exposed females exhibited significantly lower immobility duration compared to music‐exposed males, suggesting a sex‐specific improvement in adaptive behaviors that may represent improved stress resiliency in response to exposure to classical music. The current interpretation of reduced immobility representing an adaptive stress coping response is consistent with previous evidence in the prairie vole model, indicating that the behavioral state of immobility is coupled with direct physiological alterations associated with increased stress, relevant to helplessness and a depression‐like profile [73]. The present data align with previous findings, which demonstrate music prevents inflammatory, dysfunctional HPA axis‐, and maladaptive stress responses to chronic stressors, such as in the tail suspension test, elevated plus maze, and OFT in mice [55]. These data also support other research indicating that environmental treatments, such as the presence of a running wheel in the home cage, improve maladaptive coping responses in the swim task in prairie voles exposed to previous social isolation and mild environmental stressors [43]. Some behaviors observed in the OFT were also influenced by music, including increased center section exploration and increased grooming, suggesting reduced anxiety‐like responses in both sexes. Previous research indicates that increased center section exploration in the OFT is adaptive in various rodent models of stress, including prairie voles [64, 65, 92, 93]. Grooming behavior—particularly when it occurs in exposed areas of a novel environment—may indicate a lower level of perceived threat, as the animal feels safe enough to engage in self‐care rather than remain hypervigilant [24, 94, 95]. Additional behaviors in the OFT were not influenced by previous exposure to music, including exploratory motion, rearing, and escape attempts, indicating that the benefits of music may be specific to improving later stress reactivity and anxiety‐relevant behaviors rather than general exploratory behaviors or motor function.

To support adaptive behavioral responses following chronic social isolation, music has important effects on cardiovascular responses in both sexes. Male and female groups exposed to music during social isolation exhibited reduced resting HR compared to those in ambient noise conditions. Along with reduced HR, the SDNN index increased in both males and females previously exposed to music, indicating improved autonomic regulation through enhanced sympathetic and parasympathetic balance [85, 86]. The effect size for the SDNN index was slightly stronger in males versus females, which might indicate slightly improved autonomic balance in males compared to females after music exposure. Altered autonomic function is further supported by the RMSSD measure, which was significantly increased in both previous music exposure groups compared to the ambient groups, with no sex difference observed. Elevated RMSSD is hypothesized to represent enhanced parasympathetic activity and greater vagal influence on cardiac function [87, 96]. Social isolation increases the risk of cardiovascular disease via increased HR and reduced HR variability in humans [86, 97, 98], and these relationships are further supported by research in prairie voles [41, 82]. Similar physiological indices have been investigated in other studies, including rats and nonhuman primates, with comparable findings [99, 100, 101]. For example, social separation increased HR in cynomolgus monkeys, and hypertensive rats responded most favorably to Chinese classical music, showing lowered systemic blood pressure and reduced myocardial hypertrophy relative to other types of music [100, 101]. To our knowledge, this study provides the first evidence that music exposure in chronically socially isolated prairie voles alters HR and HR variability. The data shown here have translational implications for humans, supporting recent evidence that music has cardiovascular and autonomic benefits using multiple study designs [102, 103].

Further investigation of physiological changes across the study design indicates that chronic social isolation significantly increases corticosterone metabolite concentrations relative to baseline levels. After music exposure or ambient noise conditions during the final 4 weeks of social isolation, music reversed corticosterone concentrations to baseline levels while ambient noise was associated with continued elevations of corticosterone metabolite levels. This pattern of results suggests improved HPA axis function in chronically isolated prairie voles exposed to music. The HPA axis helps to maintain allostasis by regulating corticosterone secretion [104]. Chronic overactivation or stress disrupts its feedback system, such that elevated levels of corticosterone dysregulate further HPA axis communication and can damage the brain and peripheral organs [95]. Music has been associated with altered neurobiological changes [58, 105], which may help to reduce dysfunction of the HPA axis and explain the decreased corticosterone levels for those in the music group. Further, music has been shown to enhance positive effects of pharmacological interventions to aid in treating pain, depression, and other chronic medical conditions [106].

The present study provides evidence that exposure to classical music during chronic social isolation in prairie voles attenuates later adverse effects of social isolation in both sexes. Exposure to music during chronic social isolation reduced behaviors relevant to depression and anxiety, promoted HR and HR variability indicative of improved autonomic regulation of the heart, and reduced corticosterone metabolite concentrations equivalent to baseline levels. The neural mechanisms that underlie these improvements might involve modulation of brain networks associated with emotional regulation, cognition, and autonomic and endocrine regulation, which, therefore, might have delayed benefits on both behavior and physiology. In rodent models of stress, music exposure has improved HPA axis regulation, reduced oxidative stress and inflammation, altered neural spine density, and altered genes and growth factors relevant to neural plasticity [55, 56, 107, 108]. Music's activation of brain regions such as the default mode network has been linked to enhanced cognitive function and emotional well‐being in humans, and auditory sensory processes during music listening alter both autonomic regulation and cortical activity in the brain [47, 109]. However, the specific neural pathways and physiological processes during or following music listening that promote altered behavior, autonomic function, and endocrine function in a social context require further investigation.

In conclusion, the current study extends our knowledge about the benefits of music listening by demonstrating several improvements in behavior and physiology following chronic social isolation in a novel social rodent model. This research demonstrates that listening to classical music pieces during chronic social isolation—including a variety of volumes, tones, tempos, and instruments—alters later behaviors relevant to depression and anxiety, improves autonomic balance and cardiac function, and reduces corticosterone metabolite levels in both female and male prairie voles. However, it is important to note that this study was not designed to evaluate whether one specific type of music has a greater benefit than others or whether nonrhythmic sounds similarly alter behavior and physiology. Variations in rhythm and tempo might have unique effects on behavioral and neurobiological variables in humans and rodents [109, 110], but future research will benefit from comparing responses to specific musical sounds as well as nonmusic sounds of different frequencies [59, 110, 111]. Additionally, minor sex differences observed here might suggest that music has differential benefits in female and male rodents; however, further research is necessary to determine whether sex‐specific responses exist in other behavioral or neurobiological responses to music or nonmusic sounds. It will also be important to directly investigate whether the benefits of music interact with gender or gender identity in humans. Finally, a more detailed analysis of neural mechanisms that underlie the protective influence of music on both immediate and delayed behavioral and physiological outcomes will provide a foundation for designing therapeutic environmental strategies for social stressors in humans. Additional mechanistic research involving valid, reliable, and translational animal models will, therefore, improve our understanding of the benefits of music for humans who experience social isolation or loneliness, and inform the design of evidence‐based music intervention protocols.

## Author Contributions

All authors contributed significantly to the research and the associated manuscript, and all authors accept responsibility for the integrity of the information presented here. Yessenia Chavez and Nathan Campbell: Acquisition of data; analysis and interpretation of data; drafting and revising the manuscript; approval of final version of submitted manuscript. Yujun Liu, Hongdao Meng, and Jamie Mayer: Funding; conceptualization and design; interpretation of data; revising the manuscript; approval of final version of submitted manuscript. Christopher Wright, Alex Amidei, and Insha Butail: Acquisition of data; drafting and revising the manuscript; approval of final version of submitted manuscript. Chloe Steffel and Chirantana Dayanandaswamy: Drafting and revising the manuscript; approval of final version of submitted manuscript. Angela J. Grippo: Funding; conceptualization and design; acquisition of data; analysis and interpretation of data; drafting and revising the manuscript; approval of final version of submitted manuscript.

## Conflicts of Interest

The authors have no conflicts of interest to declare.

## Data Availability

Data for the project can be obtained here: https://osf.io/2upez/. Audio clips will be made available upon request.
